# Effect of Carbonated Steel Slag Powder on the Rheological Properties and Printability of 3D Printed Cement Composites

**DOI:** 10.3390/ma19143149

**Published:** 2026-07-22

**Authors:** Yilin Wang, Xingyu Qu, Junyu Wang, Xingang Xu, Yan Zheng, Heyang Wu, Mingxu Chen

**Affiliations:** 1 College of Civil Engineering & Architecture, Qingdao Agricultural University, Qingdao 266109, China; 19236131816@163.com (Y.W.); xyqu2002@163.com (X.Q.); 15064188200@163.com (J.W.); chenmx@qau.edu.cn (M.C.); 2 State Key Laboratory of Silicate Materials for Architectures, Wuhan University of Technology, Wuhan 430070, China; xuxingang@whut.edu.cn

**Keywords:** 3D printing, carbonation, steel slag, rheological properties, mechanical properties

## Abstract

The insufficient shape stability and uncontrollable rheological behavior of 3D printed cement composites (3DPCCs) still limit their wider application in construction. In this study, graded carbonated steel slag powder (CSS1 and CSS2) was used to adjust the rheological properties of 3DPCCs based on the oscillation shear and controlled shear-rate protocols, providing a sustainable and novel strategy for improved printability and mechanical properties. The results showed that the incorporation of carbonated steel slag into 3DPCCs effectively improved the viscoelasticity and yield behavior. As the carbonation time increased from 0 d to 21 d, the dynamic yield stresses of 3DPCCs with CSS1 and CSS2 increased from 126.08 Pa and 116.38 Pa to 707.29 Pa and 509.29 Pa, respectively. In addition, the structural deformation of 3DPCCs with CSS1 and CSS2 decreased from 20.35% and 21.61% to 9.83% and 11.29%, respectively, while the 3-day compressive strength increased from 8.5 MPa and 6.0 MPa to 14.2 MPa and 13.2 MPa, respectively. In conclusion, the carbonated steel slag shows great application potential in improving the rheological behavior and printability of 3D-printed cement composites.

## 1. Introduction

In the construction field, 3D printing technology enables layer-by-layer, mold-free forming of complex components based on digital models. The typical forming process mainly includes paste pumping, extrusion, and layer stacking [1,2,3]. To achieve satisfactory printability, the paste needs to have sufficient fluidity during the extrusion stage and sufficient stiffness to ensure structural stability after extrusion. Rheological properties and printability are the key factors determining the printing quality and structural deformation of 3DPCCs. The synergistic effect directly affects the smoothness of the printing process and the stability of the formed structure [4,5,6].

For 3DPCCs, rheological parameters such as static yield stress, dynamic yield stress, plastic viscosity, thixotropy, and structural build-up are closely related to extrudability and buildability [7,8]. Static yield stress mainly determines the shape-retention ability of the deposited paste, while dynamic yield stress and plastic viscosity affect the continuity and stability of extrusion [9]. A relatively low yield stress is beneficial for smooth extrusion, but it may lead to excessive deformation or collapse after deposition. In contrast, an excessively high yield stress may cause nozzle blockage and discontinuous filaments [10]. In addition, thixotropic recovery and structural build-up after extrusion are essential for supporting subsequently deposited layers [11]. For example, Su et al. suggested that an appropriate yield stress is beneficial to inhibiting paste flow, while a reasonable plastic viscosity can ensure the uniformity and continuity of extrusion filament [12]. Therefore, revealing the relationship between rheological properties and printability can provide guidance for improving the structural stability of 3DPCCs.

Steel slag, a large-volume solid waste produced during steelmaking, has significant potential for use in cement composites. The sustainable use of steel slag remains relatively low, and the long-term stockpiling has caused serious resource waste and environmental pressure [13]. Zhao et al. optimized the mix design of magnesium potassium phosphate cement composites and investigated its effects on the rheological properties of extrusion-based 3D-printing materials [14]. Chen et al. reported that tartaric acid significantly affected the printability, rheological behavior, and mechanical properties of 3D-printed sulphoaluminate cement paste [15]. Although steel slag contains calcium silicate and calcium hydroxide that can accelerate cement hydration, its hydration activity is restricted by the relatively low contents of active minerals such as C_3_S and C_2_S, together with the high proportions of inert phases such as Fe_3_O_4_ and RO phases [16,17]. In recent years, carbonation treatment has been regarded as an effective way for improving the reactivity and stability of steel slag. Beyond these performance-related benefits, carbonation also provides a potential route for steel slag resource utilization and CO_2_ mineralization. Reactive calcium-bearing phases in steel slag can react with CO_2_ to form stable CaCO_3_, thereby contributing to the fixation of CO_2_ in solid products [17]. Zhong et al. proposed a CO_2_-driven additive manufacturing method for steel slag mortars, finding that steel slag could act as the main reactive component in 3D-printed construction materials [18]. Oh et al. incorporated carbonated steel slag fine aggregate into 3DPCCs, finding that it could replace natural fine aggregate and provide great potential for sustainable 3DPCCs [19]. Furthermore, carbonation time is a key factor affecting the modification effect of steel slag, including the carbonation degree of calcareous materials, the particle surface structure, and the hydration behavior in cement materials [20]. Therefore, the incorporation of steel slag with different carbonation times may affect the rheological properties and printability of the paste.

Although previous studies have investigated rheology regulation, steel slag utilization, and carbonation treatment in cementitious materials, the effects of carbonation duration and particle-size differences in steel slag on the rheological build-up, printability, and mechanical performance of 3DPCCs remain insufficiently understood. In this study, two types of steel slag with different carbonation times were incorporated into 3DPCCs, aiming to regulate the rheological properties and improve the printability of the paste. The oscillation shear and controlled shear-rate protocols were applied to characterize the structural build-up of the paste. Carbonated steel slag with different particle sizes and carbonation times was used to inspect the rheological evolution and printability of 3DPCCs. The results provide a basis for controllable rheology and the efficient utilization of steel slag in sustainable 3DPCCs.

## 2. Materials and Methods

### 2.1. Raw Materials

**Figure 1:**
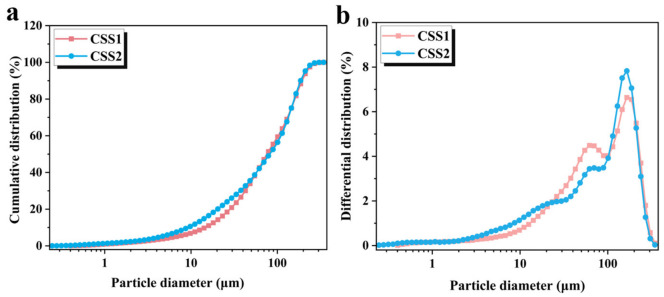
Particle size distribution of steel slag: (**a**) cumulative distribution and (**b**) differential distribution.

**Figure 2:**
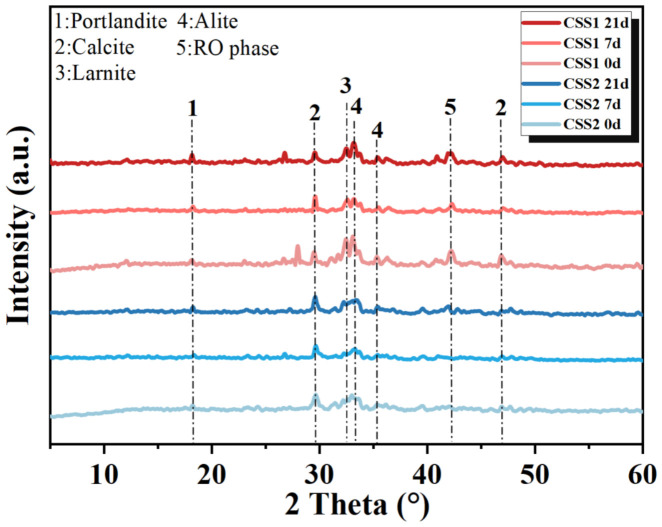
Effects of carbonation time on the XRD patterns of steel slag powders.

Ordinary Portland cement (OPC, Grade 42.5, China United Cement Co., Ltd., Beijing, China), with a density and Blaine specific surface area of approximately 3.10 g/cm^3^ and 340 m^2^/kg, respectively, bentonite (Macklin Biochemical Technology Co., Ltd., Shanghai, China), hydroxypropyl methyl cellulose (HPMC, Heda Co., Ltd., Zibo, China), water reducing agent (WRA, polycarboxylate-based, Shandong Provincial Academy of Building Research, Jinan, China), calcium formate (CF, Macklin Biochemical Technology Co., Ltd., Shanghai, China), and quartz sand were used in this study. Two types of converter steel slag were selected for the experiment: SS1 from Rizhao Iron and Steel Group; SS2 from Laiwu Iron and Steel Group. The chemical compositions of OPC and steel slag powders are shown in Table 1. The particle-size distributions of the steel slag powders are presented in Figure 1. Based on the XRD patterns in Figure 2, the mineralogical compositions of SS1 and SS2 were analyzed to identify the main crystalline phases, including portlandite, calcite, larnite, alite, and the RO phase.

### 2.2. Preparation Process of Carbonated Steel Slag

The raw steel slag was first crushed and then divided into six groups according to the carbonation duration, including 0, 1, 3, 7, 14, and 21 d. The uncarbonated steel slag was used as the reference sample.

The carbonation treatment was conducted in a carbonation chamber under a temperature of 25 ± 1 °C and a gas flow rate of 0.5 L/min. After reaching the designated carbonation duration, the steel slags were removed from the chamber. Finally, the steel slag powder was sealed, labeled, and stored for subsequent experiments.

**Figure 3:**
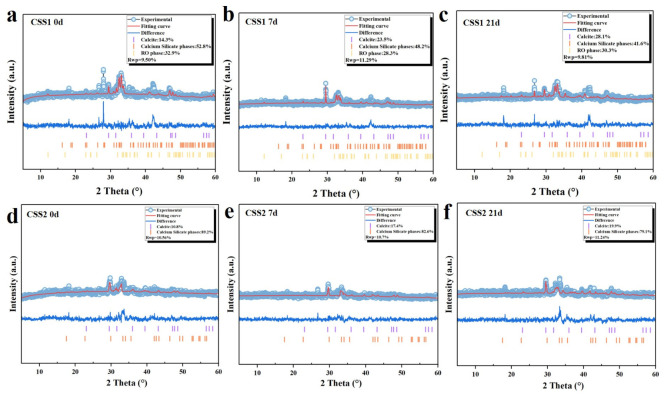
Effects of carbonation time on the Rietveld refinement results of steel slag powders: (**a**) CSS1 0 d, (**b**) CSS1 7 d, (**c**) CSS1 21 d, (**d**) CSS2 0 d, (**e**) CSS2 7 d, and (**f**) CSS2 21 d.

To further quantify the carbonation degree of steel slag, Rietveld refinement was performed on the XRD patterns of CSS1 and CSS2, and the relative content of calcite was used as a semi-quantitative indicator. As shown in Figure 3, with the carbonation time increasing from 0 d to 21 d, the calcite content in CSS1 increased from 14.3% to 28.1%, while that in CSS2 increased from 10.8% to 19.9%. This indicates that prolonged carbonation promoted the formation of CaCO_3_, and CSS1 exhibited a more pronounced carbonation response.

### 2.3. Preparation Procedures

The 3DPCCs were prepared as follows, with the mix proportions given in Table 2.

(1) Firstly, OPC, bentonite, quartz sand, and steel slag were dry-mixed for 2 min to form a solid blend;
(2) Secondly, a liquid mixture of WRA, HPMC, and CF was prepared by dissolving them in water, and then it was stirred together with the solid mixture for 2 min;
(3) Thirdly, the prepared paste was placed into the extrusion device, and the composites were fabricated using the 3D printing equipment;
(4) Finally, the specimens were kept at 20 °C and 90% relative humidity for 72 h.

### 2.4. Test Methods

#### 2.4.1. Particle Size Distribution

The size distributions of CSS1 and CSS2 were measured with a LS13320 (Beckman Coulter, Inc., Brea, CA, USA) particle size analyzer.

#### 2.4.2. 3D Printing Parameters

A 3D printing system (Nanjing Geqi Machinery Equipment Co., Ltd., Nanjing, China) was used in this study. The designed dimensions of the printed specimens were 370 mm × 210 mm × 45 mm. During printing, the translational speed was maintained at 8 mm/s. The same printing path was adopted for all mixtures to ensure comparability of the experimental results.

#### 2.4.3. Rheological Properties

The viscoelastic response and yield stress behavior of the 3DPCCs were evaluated with a rotational rheometer (HAAKE Mars 40, Thermo Fisher Scientific, Waltham, MA, USA).

The viscoelastic behavior was evaluated using two oscillatory tests: stress sweep and strain sweep protocols. During the stress sweep test, the oscillation frequency was maintained at 1 Hz, and the shear stress was gradually increased from 0.1 to 1300 Pa. The initial stable region of the elastic modulus (G′) curve was regarded as the linear viscoelastic region (LVR), indicating that structural damage within this shear stress range was recoverable. In the strain sweep protocol, the frequency was fixed at 1 Hz, and the strain was increased gradually from 10^−6^ to 10%. The variations in G′, G″, and phase angle (δ) were recorded, aiming to evaluate the elastic response and deformation resistance of the paste under different deformation levels.

The static and dynamic yield behaviors were evaluated using a controlled shear-rate protocol. In the static yield behavior test, the sample was initially conditioned at 50 s^−1^ for 2 min, rested for 10 min, and then sheared at 0.1 s^−1^ for 2 min. The maximum stress recorded during this stage was defined as the static yield stress to characterize the post-extrusion shape stability. For the dynamic yield behavior test, the paste was also pre-sheared at 50 s^−1^ for 2 min and then allowed to rest for 2 min, followed by a shear-rate ramp from 0 to 50 s^−1^ within 2 min and then back to 0 [21].

#### 2.4.4. Structural Deformation

In this study, dimensional deformation was used to assess the shape stability of the printed specimens. After extrusion, the measured dimensions were recorded and compared with the target dimensions. The deformation index was calculated using the following equation: $Z\;=\;\frac{\left|L-L_{0}\right|}{{3L}_{0}}+\frac{\left|W-W_{0}\right|}{{3W}_{0}}+\frac{\left|H-H_{0}\right|}{{3H}_{0}}\;\times \;100\%$, where Z represents the structural deformation; W_0_, L_0_, and H_0_ denote the dimensions of the specimen; and W, L, and H correspond to the greatest dimensions of the printed specimen. The deformation degree of the printed structure is comprehensively characterized by the dimensional deviations in each direction.

#### 2.4.5. Mechanical Properties

The flexural and compressive strength of 3DPCCs were evaluated by using a universal testing machine (MTS Systems Corporation, Eden Prairie, MN, USA). Prismatic specimens of 40 mm × 40 mm × 160 mm were cured at 20 °C and 90% relative humidity for 72 h after printing. The loading rate was 0.3 kN/s. For each mixture, three parallel specimens were tested.

#### 2.4.6. X-Ray Diffraction (XRD)

XRD (D8-Advance, Bruker, Karlsruhe, Germany) was used for phase composition analysis. XRD patterns were collected using Cu Kα radiation over a 2θ range of 5–60°, with a step size of 0.02° and a counting time of 0.1 s per step. For the carbonated steel slag powders, Rietveld refinement was further performed to quantify the relative contents of crystalline phases.

#### 2.4.7. Scanning Electron Microscopy (SEM)

The microstructure of the 3DPCCs was observed by SEM (Hitachi Regulus 8100, Tokyo, Japan).

## 3. Results and Discussion

### 3.1. Viscoelasticity: Shear Stress Sweep Protocol

**Figure 4:**
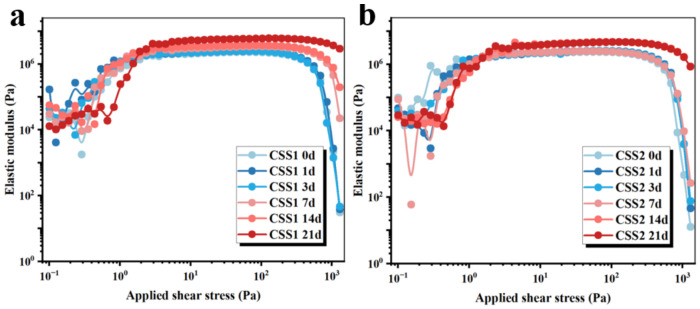
Effects of carbonation time on the elastic modulus of 3DPCCs under stress sweep protocol: (**a**) CSS1, (**b**) CSS2.

The elastic modulus reflects the ability of the paste to store elastic energy during oscillatory shear and can be used to evaluate the viscoelasticity and structure stability of 3DPCCs [22]. In Figure 4, the initial plateau region of the storage modulus curve was identified as the LVR. Upon the applied shear stress exceeding the transition point, the elastic modulus decreased sharply, revealing the breakdown of the flocculated structure. As the carbonation time increased from 0 d to 21 d, the elastic modulus of 3DPCCs with CSS1 and CSS2 increased significantly, indicating that carbonation enhanced interparticle interactions within the paste and improved the structural stiffness of the paste. The improvement in 3DPCCs with CSS1 was more pronounced than that in 3DPCCs with CSS2. The reason is that the finer particle size distribution of SS1 promoted the carbonation process of calcium-rich phases in the steel slag, leading to the generation of more carbonation products and increasing the stiffness of the paste.

**Figure 5:**
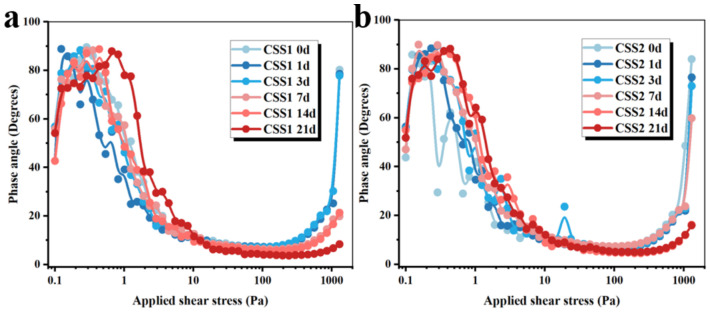
Effects of carbonation time on the phase angle of 3DPCCs under the stress sweep protocol: (**a**) CSS1 and (**b**) CSS2.

**Figure 6:**
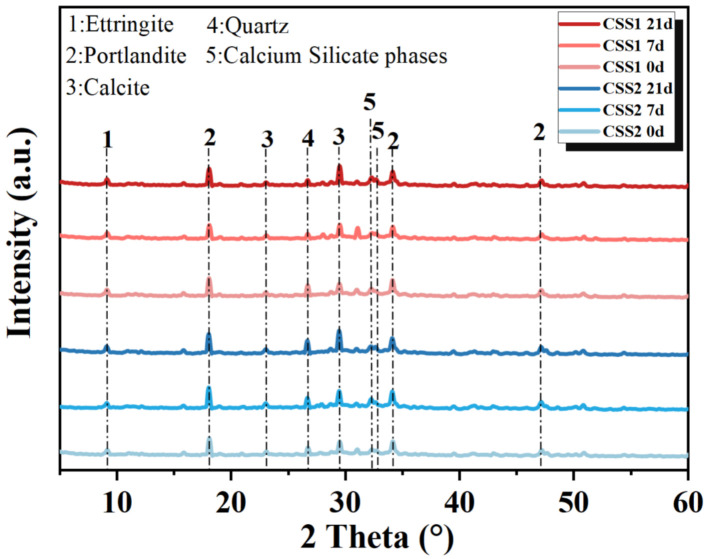
Effects of carbonation time on the XRD patterns of 3DPCCs with CSS1 and CSS2.

**Figure 7:**
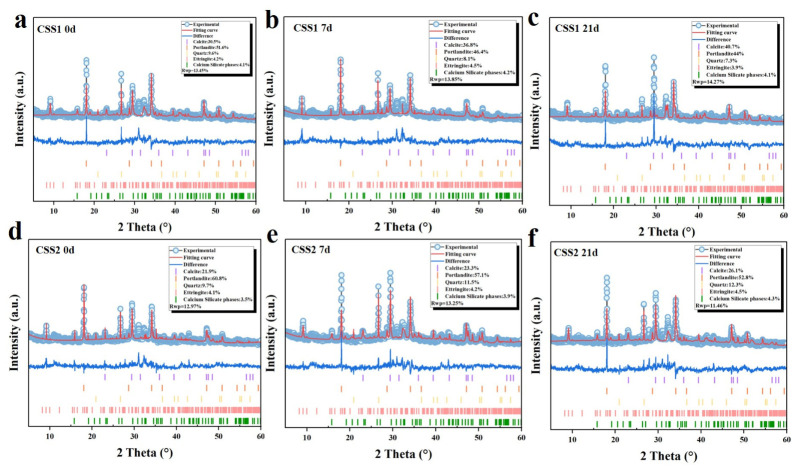
Effects of carbonation time on the Rietveld refinement results of 3DPCCs: (**a**) CSS1 0 d, (**b**) CSS1 7 d, (**c**) CSS1 21 d, (**d**) CSS2 0 d, (**e**) CSS2 7 d, and (**f**) CSS2 21 d.

When the phase angle is below 45°, the paste exhibits elastic-dominated behavior [23]. In Figure 5, the phase angles of 3DPCCs with CSS1 and CSS2 decreased rapidly with increasing shear stress and remained below 45°, revealing that both types of 3DPCCs exhibit obvious solid−like behavior. Under high shear stress, the phase angles of 3DPCCs with CSS1 and CSS2 at shorter carbonation times increased significantly, reflecting that the structures of the paste were damaged and gradually changed into a viscous flow state. As the carbonation time increased from 0 d to 21 d, the phase angle of 3DPCCs with CSS1 was always lower than that of 3DPCCs with CSS2, suggesting that 3DPCCs with CSS1 exhibited a higher elastic response and better structural stability. To further clarify the phase changes associated with the viscoelastic behavior of the paste, XRD analysis and Rietveld refinement were performed. As shown in Figure 6, the intensity of the CaCO_3_ peaks generally increased with increasing carbonation time, and the peaks of 3DPCCs with CSS1 were more pronounced than those of 3DPCCs with CSS2. This trend was further confirmed by the Rietveld refinement results shown in Figure 7. As the carbonation time increased from 0 d to 21 d, the calcite content increased from 30.5% to 40.7% in 3DPCCs with CSS1 and from 21.9% to 26.1% in 3DPCCs with CSS2. The greater increase in calcite content in 3DPCCs with CSS1 may be related to the finer particle-size distribution of SS1, which provided more contact interfaces for carbonation. The higher calcite content was also consistent with the lower phase angle and more pronounced solid-like behavior of 3DPCCs with CSS1.

**Figure 8:**
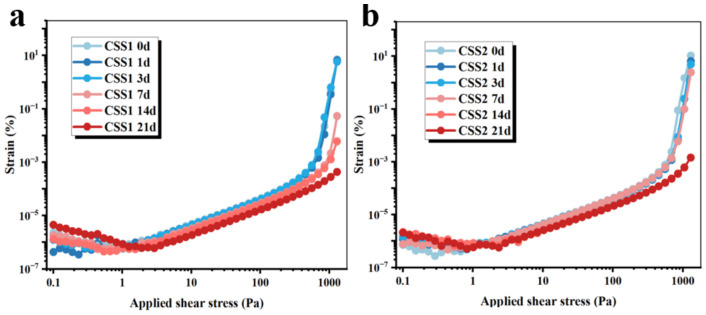
Effects of carbonation time on the total strain of 3DPCCs under the stress sweep protocol: (**a**) CSS1 and (**b**) CSS2.

Figure 8 shows the effects of 3DPCCs with CSS1 and CSS2 at different carbonation times on the total strain of 3DPCCs under the oscillation shear stress sweep protocol. The strain response of the composites decreased notably with prolonged carbonation time. The incorporation of SS1 reduced the dimensional change in the composites under loading more effectively than SS2. Lower structural deformation in the paste favors the build−up of 3D-printed samples.

### 3.2. Viscoelasticity: Shear Strain Sweep Protocol

**Figure 9:**
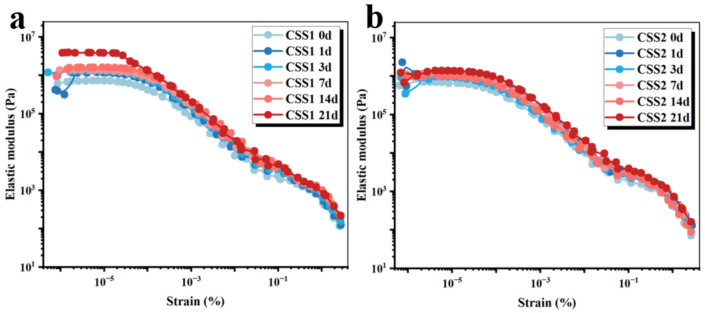
Effects of carbonation time on the elastic modulus of 3DPCCs under the strain sweep protocol: (**a**) CSS1 and (**b**) CSS2.

The strain sweep protocol was used to characterize the elastic behavior of 3DPCCs and monitor the evolution of elastic modulus with shear strain [24]. Figure 9 shows the elastic modulus of 3DPCCs with CSS1 and CSS2 at different carbonation times. At low strain levels, the elastic modulus of both groups remained relatively stable. As the shear strain increased, the elastic modulus gradually decreased, indicating that the internal flocculation network structure of the paste was damaged. The paste gradually changed from an elastic-dominated state to a viscous flow state. With the carbonation time increasing from 0 to 21 d, the elastic modulus of 3DPCCs with CSS1 and CSS2 increased significantly. The improvement in paste buildability was more pronounced for 3DPCCs with CSS1.

**Figure 10:**
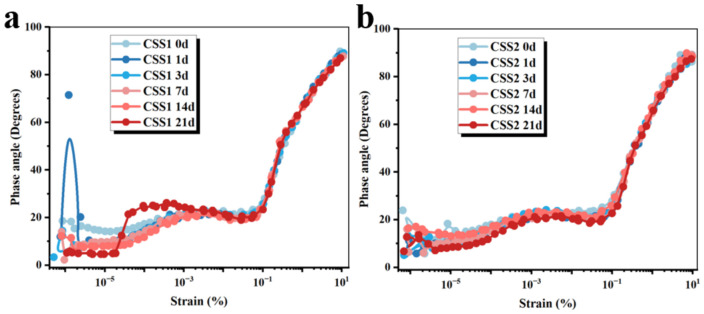
Effects of different carbonation times on the phase angle of 3DPCCs under the strain sweep protocol: (**a**) CSS1 and (**b**) CSS2.

In Figure 10, with increasing steel slag carbonation time, the phase angles of 3DPCCs with CSS1 and CSS2 generally decreased, and the stable region with phase angles lower than 45° gradually broadened. This suggested that the paste exhibited a stronger elastic response, which contributed to improved structural stability and buildability. Compared with 3DPCCs with CSS2, 3DPCCs with CSS1 exhibited a lower phase angle and a smaller fluctuation range, showing a higher elastic-dominated behavior and a more stable internal structure. With further extension of carbonation time, the phase angle of 3DPCCs with CSS1 decreased more significantly, indicating that 3DPCCs with CSS1 had better resistance to shear disturbance and higher shape-retention ability than 3DPCCs with CSS2. In contrast, 3DPCCs with CSS2 exhibited a higher phase angle at the early carbonation stage, reflecting a higher viscous response and relatively weaker structural stability. The results show that incorporation of SS1 promoted the formation of a stronger elastic structure, resulting in improved buildability.

### 3.3. Static Yield Stress

**Figure 11:**
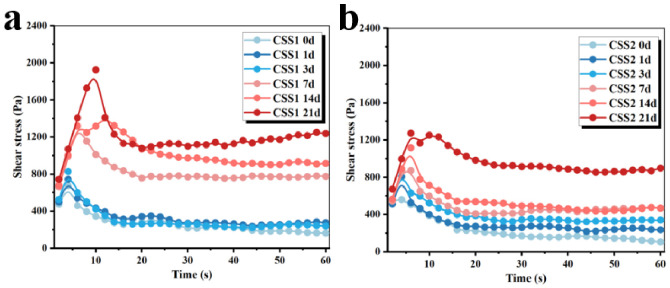
Effects of carbonation time on the shear stress of 3DPCCs at a low shear rate: (**a**) CSS1 and (**b**) CSS2.

**Figure 12:**
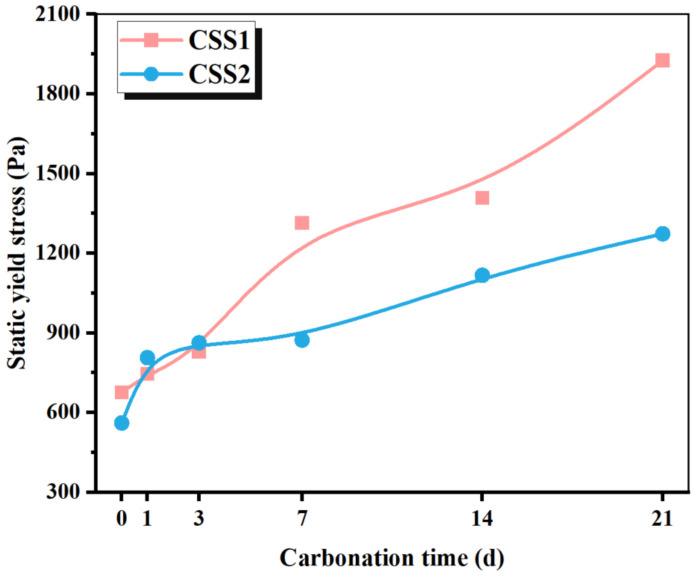
Effects of carbonation time on the static yield stress of 3DPCCs.

Static yield stress is an index for assessing the buildability of 3DPCCs, indicating the shape retention and deformation resistance of the paste [25]. As illustrated in Figure 11, the shear stress of the paste first increased rapidly to the extreme value and then decreased gradually with time until reaching a stable state. As illustrated in Figure 12, the extreme shear stress was treated as the static yield stress, representing the critical stress required to break down the initial cementitious structure of the paste. As the carbonation time increased from 0 d to 21 d, the static yield stresses of 3DPCCs with CSS1 and CSS2 increased from 675 Pa and 560 Pa to 1925 Pa and 1273 Pa, respectively. This indicates that carbonation treatment enhanced the initial cementitious structure and deformation resistance of the paste. The improvements in interlayer stacking and shape retention were more pronounced for 3DPCCs with CSS1. Such a result is similar to the research of Yuan et al., who found that limestone powder or CaCO_3_-rich fillers can affect the rheological behavior of cement pastes by changing particle packing and interparticle forces [26].

### 3.4. Dynamic Yield Stress

**Figure 13:**
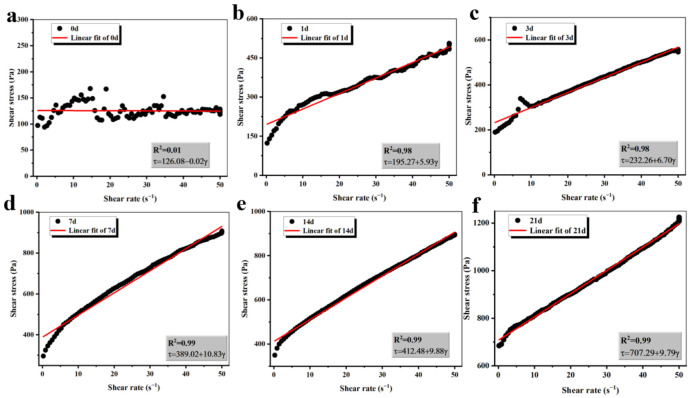
Effects of carbonation time on the dynamic yield stress and plastic viscosity of 3DPCCs: (**a**) CSS1 0d, (**b**) CSS1 1 d, (**c**) CSS1 3 d, (**d**) CSS1 7 d, (**e**) CSS1 14 d, (**f**) CSS1 21 d.

**Figure 14:**
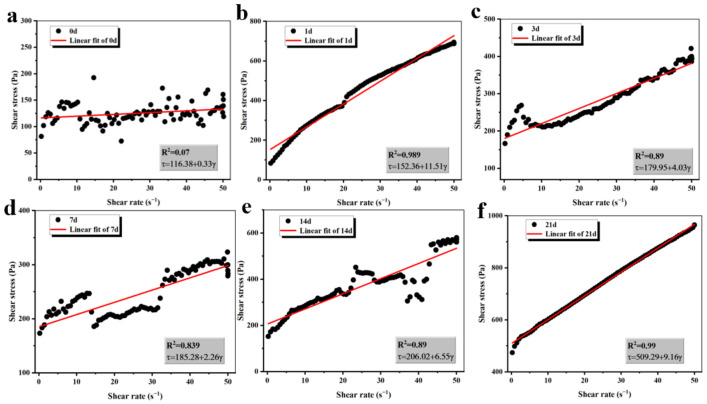
Effects of carbonation time on the dynamic yield stress and plastic viscosity of 3DPCCs: (**a**) CSS2 0d, (**b**) CSS2 1 d, (**c**) CSS2 3 d, (**d**) CSS2 7 d, (**e**) CSS2 14 d, (**f**) CSS2 21 d.

Dynamic yield stress represents the stress required for the paste to start flowing and significantly affects the extrusion capacity and forming stability of 3DPCCs [27]. Maintaining an appropriate dynamic yield stress is crucial for ensuring good extrusion fluidity and interlayer shape stability of 3DPCCs. The Bingham model was used to fit the shear stress curves under variable shear rates. Figure 13 and Figure 14 show the influence of carbonation time on the shear stress of 3DPCCs with CSS1 and CSS2 at variable shear rates, respectively. When the carbonation time increases from 0 d to 21 d, the dynamic yield stresses increased from 126.08 to 707.29 Pa for 3DPCCs with CSS1 and from 116.38 to 509.29 Pa for 3DPCCs with CSS2. Relative to 3DPCCs with CSS2, 3DPCCs with CSS1 exhibited higher dynamic yield stress and plastic viscosity, indicating greater flow resistance of the composites. In addition, the R^2^ values exceeded 0.89, suggesting that the Bingham model was suitable for describing the shear stress and shear rate relationship of the paste.

### 3.5. Structural Deformation

**Figure 15:**
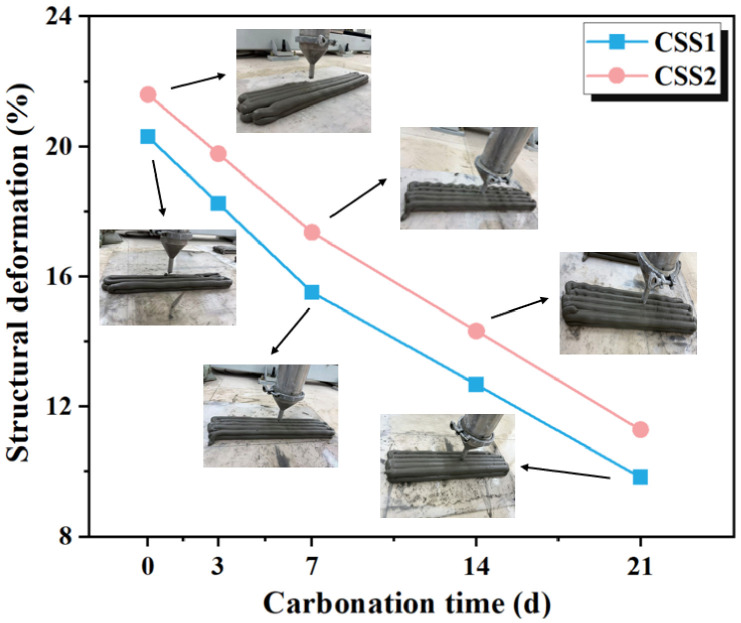
Effects of carbonation time on the deformation behavior of 3DPCCs.

**Figure 16:**
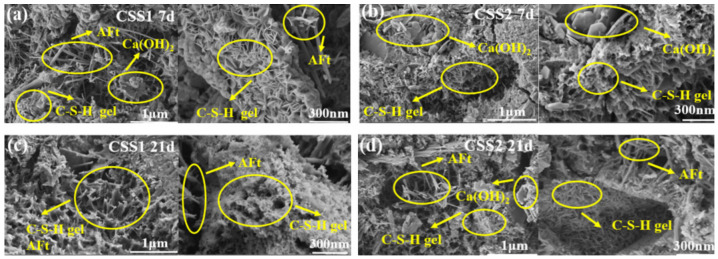
Effects of carbonation time on the SEM micrographs of 3DPCCs: (**a**) CSS1 7 d, (**b**) CSS2 7 d, (**c**) CSS1 21 d, (**d**) CSS2 21 d.

A lower structural deformation indicates that the printed specimens possess better shape retention ability during the layer-by-layer stacking process, which is beneficial for ensuring the subsequent mechanical properties [28]. As illustrated in Figure 15, the deformation rate of 3DPCCs with CSS1 is lower than that of 3DPCCs with CSS2 at the same carbonation time, showing that the incorporation of SS1 is more effective in enhancing the shape stability of printed structures. Meanwhile, as the carbonation time increased from 0 d to 21 d, the structural deformations of 3DPCCs with CSS1 and CSS2 decreased from 20.35% to 9.83% and 21.61% to 11.29%, respectively. This indicates that carbonation treatment helps improve the shape stability and buildability of printed structures. The SEM observations further confirmed the microstructural basis for the improvement in structural stability. As shown in Figure 16, needle-like and flocculent hydration products were observed in 3DPCCs with CSS1 after 7 d of carbonation and were tentatively identified as AFt and C-S-H gel, respectively. When the carbonation time was extended to 21 d, the needle-like crystals became more interlaced, the number of flocculent products increased, and the overall microstructure appeared denser. In contrast, the hydration products in 3DPCCs with CSS2 remained relatively dispersed at 21 d, with more visible pores. Meanwhile, the finer particle-size distribution provided more reactive interfaces for CO_2_ diffusion and reaction, resulting in the formation of more carbonation products and a higher paste stiffness. This observation agrees with the findings reported by Mo et al. [29] on the mechanical behavior and microstructural evolution of calcium carbonate binders derived from carbonated steel slag paste, indicating that carbonation can promote the formation of CaCO_3_, thereby enhancing interfacial bonding, improving microstructural densification, and increasing the structural integrity of cement composites.

### 3.6. Mechanical Strength

**Figure 17:**
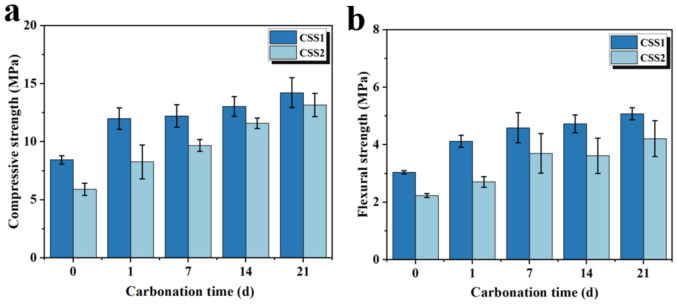
Effects of carbonation time on mechanical properties of 3DPCCs: (**a**) compressive strength and (**b**) flexural strength.

Mechanical properties are essential parameters for assessing the performance of 3DPCCs [30]. Figure 17a shows that the compressive strengths of 3DPCCs with CSS1 and CSS2 increase with prolonged carbonation. As the carbonation time increased from 0 d to 21 d, the compressive strengths of 3DPCCs with CSS1 and CSS2 increased from 8.5 MPa and 6.0 MPa to 14.2 MPa and 13.2 MPa, respectively. Compared with 3DPCCs with CSS2, 3DPCCs with CSS1 exhibited higher compressive strength at each carbonation time, suggesting a more pronounced effect on improving structural densification and mechanical performance. As illustrated in Figure 17b, the flexural strength of 3DPCCs with CSS1 and CSS2 increased with prolonged carbonation time. As the carbonation time increased from 0 d to 21 d, the flexural strengths of 3DPCCs with CSS1 and CSS2 increased from 3.0 MPa and 2.2 MPa to 5.1 MPa and 4.2 MPa, respectively. Compared with 3DPCCs with CSS2, 3DPCCs with CSS1 exhibited higher flexural strength at each carbonation time, indicating better internal structural continuity and greater resistance to bending failure. This may be attributed to the formation of carbonation products, such as CaCO_3_, during the carbonation process. These products can fill pores and enhance the interfacial bonding between hydration products and particles, thereby densifying the microstructure. This result is consistent with previous studies on carbonated steel slag and CaCO_3_-bearing cementitious materials. The strength enhancement observed in this study agrees with the findings of He et al. [31], who attributed the improved compressive strength of mortar containing carbonated BOF slag to CaCO_3_ formation and increased amounts of C-S-H and AFt. Similarly, Srivastava et al. [32] reported that carbonated steel slag contributed to a denser microstructure with fewer micropores. These findings indicate that carbonated steel slag can improve the mechanical performance of 3DPCCs while providing a potential route for the value-added utilization of steel slag.

## 4. Conclusions

In this study, two types of carbonated steel slag powders with different particle sizes were incorporated into 3DPCCs. The utilization of carbonated steel slag presents great potential for developing the composites. The major findings are summarized as follows:

(1) Both carbonated SS1 and SS2 improved the viscoelastic behavior of 3DPCCs. As the carbonation time increased from 0 d to 21 d, the elastic modulus increased, and the phase angle decreased for 3DPCCs with CSS1 and CSS2.
(2) The two types of carbonated steel slag led to a significant increase in the yield value of 3DPCCs. With the increase of carbonation time from 0 d to 21 d, the static yield stresses of 3DPCCs with CSS1 and CSS2 increased from 675 and 560 Pa to 1925 and 1273 Pa, respectively. The dynamic yield stresses also increased from 126.08 and 116.38 Pa to 707.29 and 509.29 Pa.
(3) The two types of carbonated steel slags effectively reduced the structural deformation rate of 3DPCCs. As the carbonation time increased from 0 d to 21 d, the structural deformation of 3DPCCs with CSS1 and CSS2 decreased from 20.35% and 21.61% to 9.83% and 11.29%, respectively.
(4) With the increase of carbonation time from 0 d to 21 d, the compressive strengths of 3DPCCs with CSS1 and CSS2 increased from 8.5 MPa and 6.0 MPa to 14.2 MPa and 13.2 MPa, respectively, while the flexural strengths increased from 3.0 MPa and 2.2 MPa to 5.1 MPa and 4.2 MPa, respectively.

## Figures and Tables

**Table 1:** Chemical composition of OPC and steel slag (% by mass).

Components	Cr_2_O_3_	SO_3_	TiO_2_	P_2_O_5_	MgO	MnO	Al_2_O_3_	SiO_2_	Fe_2_O_3_	CaO	Others
SS1	0.45	0.49	2.30	2.36	4.10	4.16	5.00	18.09	23.22	38.30	1.53
SS2	0.73	0.80	1.41	1.00	3.76	3.87	7.40	12.75	27.04	39.49	1.75
OPC	—	2.7	0.6	0.21	5.2	0.2	11.75	28.3	2.8	47.13	1.11

**Table 2:** Mix proportions of 3DPCCs (% by mass).

OPC	HPMC	WRA	CF	Bentonite	Sand	Water	CSS1	CSS2
100	0.10	0.30	1.0	1.0	50	35	10 (0, 1, 3, 7, 14, 21 d)	—
100	0.10	0.30	1.0	1.0	50	35	—	10 (0, 1, 3, 7, 14, 21 d)

Note: OPC was fixed at 100 by mass. Steel slag was added as an additional component at 10% of OPC mass, rather than replacing OPC.

## Data Availability

The original contributions presented in this study are included in the article. Further inquiries can be directed to the corresponding authors.

## References

[1] Buswell R.A., Leal de Silva W.R., Jones S.Z., Dirrenberger J. (2018). 3D printing using concrete extrusion: A roadmap for research. Cem. Concr. Res..

[2] Cui H., Li Y., Cao X., Huang M., Tang W., Li Z. (2022). Experimental Study of 3D Concrete Printing Configurations Based on the Buildability Evaluation. Appl. Sci..

[3] Chen M., Li L., Wang J., Huang Y., Wang S., Zhao P., Lu L., Cheng X. (2020). Rheological parameters and building time of 3D printing sulphoaluminate cement paste modified by retarder and diatomite. Constr. Build. Mater..

[4] Tran N., Van Tran M., Tran P., Nguyen A.K., Nguyen C.Q. (2024). Eco-friendly 3D-printed concrete using steel slag aggregate: Buildability, printability and mechanical properties. Int. J. Concr. Struct. Mater..

[5] Liu X., Sheng H., Feng B., Zhao P., Huang Y., Wang S., Sun K., Chen M., Lu L. (2024). Effect of potassium and sodium-based electrolyzed water on the rheological properties and structural build-up of 3D printed cement composites. J. Build. Eng..

[6] Alonso-Canon S., Blanco-Fernandez E., Castro-Fresno D., Yoris-Nobile A.I., Castanon-Jano L. (2024). Comparison of reinforcement fibers in 3D printing mortars using multi-criteria analysis. Int. J. Adv. Manuf. Technol..

[7] Rehman A.U., Kim J.H. (2021). 3D concrete printing: A systematic review of rheology, mix design, mechanical, microstructural, and durability characteristics. Materials.

[8] Luo J., Wang Q., Wang L., Fang M. (2026). A review of the rheological properties of 3D-printed concrete: Raw materials, printing parameters, and evolution mechanisms. Buildings.

[9] Chen M., Yang L., Zheng Y., Huang Y., Li L., Zhao P., Wang S., Lu L., Cheng X. (2020). Yield stress and thixotropy control of 3D-printed calcium sulfoaluminate cement composites with metakaolin related to structural build-up. Constr. Build. Mater..

[10] Panda B., Noor Mohamed N.A., Paul S.C., Bhagath Singh G.V.P., Tan M.J., Šavija B. (2019). The effect of material fresh properties and process parameters on buildability and interlayer adhesion of 3D printed concrete. Materials.

[11] Lu B., Weng Y., Li M., Qian Y., Leong K.F., Tan M.J., Qian S. (2019). A systematical review of 3D printable cementitious materials. Constr. Build. Mater..

[12] Su Y.L., Wu C., Shang J.Q., Zhang P. (2025). Mechanical properties of 3D-printed high-ductility cementitious composite with sulphoaluminate cement and modified crumb rubber. Constr. Build. Mater..

[13] Zhang Z.D., Jia Z.J., Jia L.T., Zhao Y.L., Wang W., Zhang Y.M., Chen Y. (2026). Research progress on rheology regulation of 3D printed concrete. J. Chin. Ceram. Soc..

[14] Zhao Z., Chen M., Xu J., Li L., Huang Y., Yang L., Zhao P., Lu L. (2021). Mix design and rheological properties of magnesium potassium phosphate cement composites based on the 3D printing extrusion system. Constr. Build. Mater..

[15] Chen M., Guo X., Zheng Y., Li L., Yan Z., Zhao P., Lu L., Cheng X. (2018). Effect of Tartaric Acid on the Printable, Rheological and Mechanical Properties of 3D Printing Sulphoaluminate Cement Paste. Materials.

[16] Zhang Z., Xiong Y., Jia Z., Cao R., Gao Y., Maruyama I., Zhang Y., Wang W. (2024). In-situ wet carbonation of steel slag powder paste made with carbonated water: Interaction mechanism between carbonation and hydration. Cem. Concr. Compos..

[17] Wang S., Li Y., Zhang J., Liu X., Wang Q. (2024). A review on the carbonation of steel slag: Properties, Mechanism, and Application. Materials.

[18] Zhong K.N., Liu Z.C., Wang F.Z., Hu S. (2024). CO_2_-driven additive manufacturing of sustainable steel slag mortars. ACS Sustain. Chem. Eng..

[19] Oh G., Oh S., Choi S. (2026). Carbonated basic oxygen furnace slag fine aggregate in concrete: Laboratory assessment and sustainable application in 3D-printed concrete components. J. Build. Eng..

[20] Liu P., Mo L., Zhang Z. (2023). Effects of carbonation degree on the hydration reactivity of steel slag in cement-based materials. Constr. Build. Mater..

[21] Peng Y., Ma K., Unluer C., Li W., Li S., Shi J., Long G. (2021). Method for calculating dynamic yield stress of fresh cement pastes using a coaxial cylinder system. J. Am. Ceram. Soc..

[22] Roussel N., Bessaies-Bey H., Kawashima S., Marchon D., Vasilic K., Wolfs R. (2019). Recent advances on yield stress and elasticity of fresh cement materials. Cem. Concr. Res..

[23] Zhang Y., Ren Q., Dai X., Tao Y., Zhang Y., Jiang Z., Van Tittelboom K., De Schutter G. (2024). A potential active rheology control approach for 3D printable cement-based materials: Coupling of temperature and viscosity modifiers. Cem. Concr. Compos..

[24] Moini R., Olek J., Zavattieri P.D., Youngblood J.P. (2022). Early-age buildability-rheological properties relationship in additively manufactured cement paste hollow cylinders. Cem. Concr. Compos..

[25] Ngo T.H., Li S., Huynh T., Zhang Y.X., Tran P. (2025). 3D printable hemp concrete: Rheological, mechanical, and microstructural properties. J. Build. Eng..

[26] Yuan H., Ma C., Liu J., Ge Z., Ling Y., Zhang H., Tawfek A.M., Sun R. (2024). The effects of particle packing and inter-particle force on rheological behaviors of binary cement paste. KSCE J. Civ. Eng..

[27] Ren Q., Xie M., Zhu X., Zhang Y., Jiang Z. (2020). Role of limestone powder in early-age cement paste considering fineness effects. J. Mater. Civ. Eng..

[28] Chen M., Yang L., Zheng Y., Li L., Wang S., Huang Y., Zhao P., Lu L., Cheng X. (2021). Rheological behaviors and structure build-up of 3D printed polypropylene and polyvinyl alcohol fiber-reinforced calcium sulphoaluminate cement composites. J. Mater. Res. Technol..

[29] Mo L., Zhang F., Deng M. (2016). Mechanical performance and microstructure of the calcium carbonate binders produced by carbonating steel slag paste under CO_2_ curing. Cem. Concr. Res..

[30] Ma L., Zhang Q., Jia Z., Liu C., Deng Z., Zhang Y. (2022). Effect of drying environment on mechanical properties, internal RH and pore structure of 3D printed concrete. Constr. Build. Mater..

[31] He D., Yang L., Guo J. (2024). Effect of carbonation degree on mineral composition, microstructure, and cementitious properties of BOF slag. J. Sustain. Metall..

[32] Srivastava S., Cerutti M., Nguyen H., Carvelli V., Kinnunen P., Illikainen M. (2023). Carbonated steel slags as supplementary cementitious materials: Reaction kinetics and phase evolution. Cem. Concr. Compos..

